# Chikungunya Disease Outbreak, Reunion Island

**DOI:** 10.3201/eid1212.060710

**Published:** 2006-12

**Authors:** Loïc Josseran, Christophe Paquet, Abdelkrim Zehgnoun, Nadège Caillere, Alain Le Tertre, Jean-Louis Solet, Martine Ledrans

**Affiliations:** * Institut de Veille Sanitaire, Saint Maurice, France;; † Cire de la Reunion, Saint Denis, France

**Keywords:** Chikungunya, Reunion, crude mortality, syndromic surveillance, letter

To the Editor: A serious outbreak of chikungunya disease recently occurred on Reunion Island (population ≈770,000) (*1*). Between March 1, 2005, and April 30, 2006, ≈255,000 cases were reported in this French territory in the Indian Ocean. Most cases occurred after mid-December 2005, with a maximum of 45,000 cases during the week of January 29 to February 4, 2006 (*2*). Surveillance figures were confirmed by a serosurvey that found a prevalence of 18% of recent infection markers in pregnant women in March 2006.

Chikungunya is a self-limiting febrile viral disease characterized by arthralgia or arthritis. Symptoms may last for several months, but recovery was, until now, considered universal (*3*). However, in January 2006, the health authorities on this island started receiving death certificates mentioning chikungunya as a cause of death, either direct or indirect. By the end of April, 213 death certificates with this finding had been received. To assess the affect of chikungunya disease, we compared the crude death rate (CDR) observed during the outbreak period with an expected death rate computed from the 2002–2004 historical data.

The study included the period January 1, 2005, through April 30, 2006. The expected number of deaths (all causes) for 2005 and 2006 was the number of deaths by sex and age observed during 2002–2004 modified by an estimation of the population size for 2005–2006. The details of this method, which was used during the heat wave in France in 2003, have been reported (*4*). The number of deaths in Reunion was obtained daily from 13 of 24 computerized registry offices throughout the island and represented 87% of the deaths on the island.

During 2005, the monthly CDR remained within expected range of statistical variation. From January through April 2006, respectively, monthly CDRs were 7.1%, 34.4%, 25.2%, and 8.3% higher than expected rates (p<0.01 for February and March). This corresponded to 226 excess deaths reported by the 13 offices participating in the study and 260 excess deaths when data were extrapolated to the entire population of the island (an increase of 18.4%) (Figure). The 260 excess deaths is a crude figure that includes potentially all causes of death. This figure leads to a rough estimate of the case-fatality rate for chikungunya disease of ≈1/1,000 cases. Excess deaths were observed mainly in persons >75 years of age.

**Figure:**
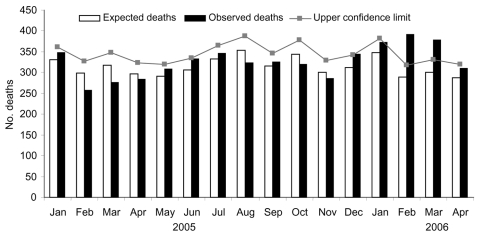
Expected and observed number of deaths reported by 13 computerized registry offices in Reunion Island, France, January 2005–April 2006.

CDRs began to exceed the expected range during the last week of January 2006 and remained elevated until the end of the study period. This situation closely matched the kinetics of the epidemic curve of chikungunya disease. CDR is a stable variable in time for a defined population. Only a massive phenomenon can have an effect on it, and no other abnormal health event affected the island at this time. Thus, the outbreak of chikungunya disease was likely responsible for most of the excess deaths observed in Reunion during the first 4 months of 2006.

Deaths associated with chikungunya disease have been rarely reported. This outbreak in Reunion is the first with such a high incidence in a setting where real-time death reporting is a standard procedure. In such settings, CDR monitoring should be considered syndromic surveillance and should be implemented when an abnormal health phenomenon affects large populations.

## References

[1] Enserink M. Infectious diseases. Massive outbreak draws fresh attention to little-known virus. Science. 2006;311:1085. 10.1126/science.311.5764.1085a 16497896

[2] Paganin F, Borgherini G, Staikowsky F, Arvin-Berod C, Poubeau P. Chikungunya on Reunion Island: chronicle of an epidemic foretold. Presse Med. 2006;35:641–6. 10.1016/S0755-4982(06)74657-7 16614609

[3] Mackenzie JS, Chua KB, Daniels PW, Eaton BT, Field HE, Hall RA, Emerging viral diseases of Southeast Asia and the Western Pacific. Emerg Infect Dis. 2001;7(Suppl):497–504. 10.3201/eid0703.010303 11485641 PMC2631848

[4] Pirard P, Vandentorren S, Pascal M, Laaidi K, Le Tertre A, Cassadou S, Summary of the mortality impact assessment of the 2003 heat wave in France. Euro Surveill. 2005;10:153–6. 16088047

